# The expression of GapA and CrmA correlates with the *Mycoplasma gallisepticum* in vitro infection process in chicken TOCs

**DOI:** 10.1186/s13567-022-01085-2

**Published:** 2022-09-02

**Authors:** Nancy Rüger, Michael P. Szostak, Silke Rautenschlein

**Affiliations:** 1 Clinic for Poultry, University of Veterinary Medicine Hannover, Hannover, Germany; 2 Institute of Microbiology, Department of Pathobiology, University of Veterinary Medicine Vienna, Vienna, Austria

## Abstract

*Mycoplasma (M.) gallisepticum* is the most pathogenic mycoplasma species in poultry. Infections cause mild to severe clinical symptoms associated with respiratory epithelial lesion development. Adherence, biofilm formation, and cell invasion of *M. gallisepticum* contribute to successful infection, immune evasion, and survival within the host. The important *M. gallisepticum* membrane-bound proteins, GapA and CrmA, are key factors for host cell interaction and the bacterial life-cycle, including its gliding motility, although their precise role in the individual infection step is not yet fully understood. In this study, we investigated the correlation between the host–pathogen interaction and the GapA/CrmA expression in an environment that represents the natural host’s multicellular compartment. We used an in vitro tracheal organ culture (TOC) model, allowing the investigation of the *M. gallisepticum* variants, Rlow, RCL1, RCL2, and Rhigh, under standardised conditions. In this regard, we examined the bacterial adherence, motility and colonisation pattern, host lesion development and alterations of mucociliary clearance. Compared to low virulent RCL2 and Rhigh, the high virulent Rlow and RCL1 were more efficient in adhering to TOCs and epithelium colonisation, including faster movement from the cilia tips to the apical membrane and subsequent cell invasion. RCL2 and Rhigh showed a more localised invasion pattern, accompanied by significantly fewer lesions than Rlow and RCL1. Unrelated to virulence, comparable mucus production was observed in all *M. gallisepticum* infected TOCs. Overall, the present study demonstrates the role of GapA/CrmA in virulence factors from adherence to colonisation, as well as the onset and severity of lesion development in the tracheal epithelium.

## Introduction

Avian mycoplasmosis is diagnosed worldwide and leads to significant economic losses in all production levels in poultry [1]. The changes in the global need for meat and egg production, increasing numbers of free-range flocks with a higher risk of mycoplasma-introduction [2], and reports of virulence reversion of circulating vaccine strains in the field [3–5] point out the need for better control- and intervention strategies. Mycoplasmas are cell wall-less bacteria and are therefore phenotypically distinct from other bacteria. Their evolutionary reduced genomic composition with a size between 580 kb and 1.380 kb [6] classifies them as the smallest self-replicating prokaryotes. The limitation of genome size makes it even more remarkable that these bacteria developed various survival strategies. Adhesion to the host cell membrane, cell invasion, antigenic- and phase variation contribute to successful immune evasion and adaption to the cellular environment [6–8]. Cytopathological changes of the host target cells, such as ciliary dysfunction, loss of epithelial integrity, and altered mucus production, may contribute to maintaining the infection process [9–11].

The most pathogenic avian mycoplasma species is *Mycoplasma* (*M.*) *gallisepticum*, the causative agent of chronic respiratory disease (CRD) in chickens and infectious sinusitis in turkeys. Reduced meat- and egg production and considerable costs for treatment and prophylactic measures are attributable to *M. gallisepticum* infections. Ciliated epithelial cells of the respiratory tract represent the main target to which *M. gallisepticum* attaches first and mediates colonisation. The ability to adhere is an essential prerequisite to initiating the infection process and is considered a major virulence factor of *M. gallisepticum* [6]. A membrane protrusion, the terminal organelle (TO), is the leading structure by which *M. gallisepticum* interacts with the host cells. The composition and formation of this organelle have been addressed in various studies and were described as clustering of cytadhesion associated- and structural proteins [12–14]. Within the *M. pneumoniae* cluster, genetic and proteomic analyses identified these proteins and found assignable homologies between the different species*,* including *M. gallisepticum* and *M. pneumoniae* [14, 15]. In *M. gallisepticum*, the primary cytadhesine GapA and the cytadherence-related molecule CrmA are necessary proteins for efficient host cell adherence and virulence [16, 17]. Cytadherence-deficient mutants, lacking the GapA and CrmA proteins, were shown to be less virulent. Trials in chicken and experiments in human lung fibroblast (MRC-5) infected with GapA/CrmA-negative variants resulted in decreased *M. gallisepticum*-detection rates, compared to the high virulent GapA/CrmA-positive variants [12, 18, 19].

Motility might be a crucial prerequisite between initial attachment and subsequent spreading and cell invasion. *M. pneumoniae* first attaches to the cilia of epithelial cells and then moves to the apical cell border, the site of cell invasion [6, 15]. *M. pneumoniae* proteins involved in this process are the cytadhesin P1 and cytadhesion-associated proteins P40/P90, the homologues of the *M. gallisepticum* proteins GapA and CrmA, respectively [14, 20, 21]. P1 has been shown to significantly contribute to motility, as observed by the gliding ability of *M. pneumoniae* on glass coverslips. After anti-P1 antibody treatment, gliding speed was significantly reduced as analysed with time-interval microscopic images [22]. P40 and P90 ensure that P1 is anchored in the bacterial membrane, which maintains the formation and function of the terminal organelle [23]. Suggestions that the homologous proteins GapA and CrmA might be involved in adherence and motility of *M. gallisepticum* [13] led to investigations of the motility of *M. gallisepticum* strain R and construction of genetic variants. As the first *M. gallisepticum* strain whose genome has been completely sequenced, strain R represents the most suitable model to investigate the infection process experimentally [24]. Low passage (Rlow) and high passage numbers (Rhigh) of the parental strain R resulted in two variants with different geno- and phenotypic characteristics [25]: the highly pathogenic GapA/CrmA-positive variant Rlow with a flask-shaped morphology and the low pathogenic GapA/CrmA-negative variant Rhigh, with its round-shaped and protrusion-less morphology [12, 18, 19]. More efficient colonisation of the trachea, higher multiplication rates and higher lesion scores in the air sacs as well as systemic spreading to inner organs were detected after infection with Rlow, which contrasts with the mild pathological changes and lower reisolation rates of Rhigh [26]. These in vivo studies point out the importance of GapA and CrmA in the colonisation and replication process. Comparable results were generated in vitro. While Rlow exhibited efficient adherence and cell invasion in human cervical adenocarcinoma (Hela-229) cells, chicken embryo fibroblasts (CEF), and MRC-5 cells, the adherence of Rhigh to these different cell types appeared to a much lower extent [17, 18, 27].

Phase variation is one of the mechanisms used by *M. gallisepticum* to evade immune defence and thereby contribute to the infection process. The well-established Rlow clonal variant RCL2 has been addressed to investigate the effects of GapA on-and-off switching on adherence and host colonisation [12, 28]. This variant lacks the GapA and CrmA proteins, which is why in vivo trials with RCL2 resulted in lower infection rates than with Rlow, but significantly higher than with Rhigh [12, 13]. Immunoblotting of organ samples taken from RCL2-infected chicken revealed a total reisolation rate of 80% from the respiratory tract and 15.7% from inner organs. Reisolations from the control group infected with Rhigh reached only 59.3% and 7.8%, respectively, indicating a higher virulence of RCL2 than Rhigh. A base substitution within the structural *gapA* gene of RCL2 exhibits a high reversion frequency [28], contrasting with the frameshift mutation’s low reversion frequency in Rhigh. This is considered to account for the different virulence of the two GapA/CrmA-negative variants, RCL2 and Rhigh.

The mucociliary clearance ensures the primary immune defence of the respiratory tract. It consists of a mucous fluid layer that is continuously transported cranially by the directed movement of the cilia. This mechanism is the first barrier that pathogens must overcome to initiate an infection in the respiratory tract. It has been shown that *M. gallisepticum* can interfere with the mucociliary clearance and thus spread more effectively. Chickens exposed to the pathogenic *M. gallisepticum* strain Ap3AS showed disintegrated epithelium, loss of cilia, and reduced intraepithelial mucous glands in the trachea [29]. In our previous study, histopathological examinations of tracheal organ cultures (TOC) confirmed the initiation of ciliary dysfunction after infection with the pathogenic *M. gallisepticum* strain S6 [30].

Based on these findings, we aimed to conduct a study in a well-controlled avian-derived in vitro model, which allows the investigation of the correlation between attachment, movement, and colonisation pattern in an environment, which represents the multicellular component of the natural host, with the expression of the proteins GapA and CrmA. The tracheal organ culture proved to be a suitable in vitro model to examine the host–pathogen-interaction of *M. gallisepticum* in the chicken respiratory tract [31, 32]. The in vivo respiratory tract situation can be very well reproduced with this organ culture system. We used the *M. gallisepticum* variants Rlow, Rhigh, RCL1 and RCL2 and looked for the bacterial infection pattern (attachment, movement, invasion, and replication) and histopathological changes, such as ciliostasis, ciliary loss, loss of epithelial integrity and altered mucus production. RCL1, the identical clone of Rlow, represents the control variant for RCL2 [28].

## Materials and methods

### Mycoplasma variants

For the present study, laboratory strains of *Mycoplasma gallisepticum* strain R variants Rlow, Rhigh, RCL1 and RCL2, were used [25, 28]. The bacteria were passaged in modified Hayflick (HFLX) medium [33] three times before plating them on Frey Agar [34]. Colonies were counted after 6 days of incubation under humid and microaerophilic conditions at 37 °C. The bacterial concentrations were determined via colony-forming unit (CFU) assay [35]. An inoculation dose of 1 × 10^3^ CFU/TOC was used for each experiment.

### Tracheal organ culture

The procedure of tracheal organ culture (TOC) preparation was described by Cherry & Taylor-Robinson [36]. Briefly, tracheas were dissected from 19 days-old specific-pathogen-free (SPF) humanely sacrificed chicken embryos (Valo BioMedia GmbH, Germany). Tracheas were stripped from connective tissue and cut into equal rings (12 / trachea). Each TOC was placed separately in a 5 mL-tube (Sarstedt AG & Co KG, Germany), filled with 1 mL 37 °C pre-warmed Medium 199 Hanks’ salts (Sigma-Aldrich), supplemented with 1% L-glutamine (200 mM, Biochrome, Berlin, Germany) and 1% Penicillin G (10 000 U/mL, Biochrome). The tubes were placed in an overhead shaker and TOCs incubated 4 days at 37 °C. After microscopic evaluation for ciliary beating, the TOCs from each embryo with a 100% activity were split equally in number and allocated to five groups.

### Pre-experiment: determination of *M. gallisepticum* growth without TOCs

The bacterial viability in organ culture medium without TOC was determined via CFU assay to exclude growth effects of the medium on the bacterial replication. Therefore, each of the four *M. gallisepticum* variants was incubated with the medium, with or without the TOCs. After one, 25, 49, 73 and 97 h post-inoculation (hpi), medium was collected from five tubes/group/time points. After serial dilution, they were plated on Frey agar in triplicates. The inoculated plates were incubated for 6 days under humid and microaerophilic conditions at 37 °C and were microscopically evaluated for colony growth. The average colony numbers per dilution were used to calculate the bacterial concentration (CFU/mL).

### Quantification of mycoplasmas adherent to TOCs via CFU assay

To determine the mycoplasma adherence rate to TOCs, *M. gallisepticum*-inoculated tracheal rings were collected after different incubation times and transferred separately into a 5 mL-tube filled with 1 mL pre-warmed (37 °C) HFLX medium and incubated for 48 h. Then, the medium of each tube was collected and used for tenfold serial dilution. Diluted medium (10 µL-drops in triplicate/dilution step/sample) was plated on Frey agar. Plate-incubation and CFU- calculation was performed as described above.

### Detection of *M. gallisepticum* antigen via immunohistochemical (IHC) staining

To determine the colonisation pattern of *M. gallisepticum* in TOCs, the bacterial antigen (Ag) was stained. The tissue sections were prepared from formalin-fixed TOCs and staining procedure was conducted as described on our previous study [30]. Primary antibodies (Abs) (polyclonal rabbit anti*-MG* [31], diluted 1:1000) were added and incubated overnight in a humid chamber at 4 °C. After washing procedures, secondary Abs were added (goat anti-rabbit IgG (H + L) labelled with Alkaline Phosphatase (AP) (Biozol Eching, Germany), diluted 1:1000) and incubated for 45 min in a humid chamber at 36 °C. To visualise the *M*. *gallisepticum* Ag-Ab-complex, the slides were stained with the VECTOR Red Alkaline Phosphatase Kit (Vector Laboratories Inc., Burlingame, CA, USA), counterstained with haemalaun and mounted with Aquatex (Merck, Darmstadt, Germany). Antigen detection was conducted microscopically (Leica Microsystems, Germany) at 400-fold and 1000-fold magnifications. Antigen-positive cells were counted, and the average number was calculated from three microscopic fields per ring with five rings/time point/group.

### DNA and RNA isolation

RNA/DNA isolation from TOCs and adherent mycoplasma was performed with the RNA/DNA purification kit from KYLT^®^ (AniCon, Germany), following the manufacturer’s instructions.

### Quantification of the *M. gallisepticum* genome and RPL13 housekeeping gene via (RT)qPCR

The bacterial genome was quantified via quantitative (q) PCR using a lipoprotein encoding gene as indicator for pathogen replication. The 60S ribosomal protein L13 (RPL13) housekeeping gene quantification was performed via reverse transcriptase (RT) qPCR [37]. The *M. gallisepticum* primer and probe design were based on the previously published sequence of *M. gallisepticum* strain R (Acc.# AY556071) [38]. Sequences for RPL13 primer and probe are accessible under Acc.# NM_204999.1 [37]. Quantitative PCR was conducted using the PerfeCTa qPCR Toughmix (2X, without ROX, Quanta) with the following cycle profile: 1 cycle 95 °C—10 min and 45 cycles: 95 °C—15 s, 60 °C—60 s. RTqPCR was performed with the qScript^™^ XLT One-Step RT-qPCR ToughMix^®^, Low ROX™ (Quanta) with the following cycling profile: 1 cycle 50 °C—10 min, one cycle 95 °C—1 min and 45 cycles 95 °C—10 s, 60 °C—40 s. The QuantStudio3 Real-Time PCR System (Applied Biosystem, Thermo Fisher Scientific) was used to conduct amplification reactions. Standard curves of the *M. gallisepticum* stocks and negative TOCs (RPL13 housekeeping gene) were generated to verify the quality of the isolation procedure and qPCR protocol. Samples were tested in duplicates per reaction. Data are presented as cycle threshold (C_T_) values, which were normalised against the RPL13 housekeeping gene of the respective sample (*Δ*C_*T*_).

### Observation for ciliary dysfunction via ciliostasis assay

TOCs were observed for ciliary activity via an inverted microscope (Zeiss, Germany) to show whether the *M. gallisepticum* R variants induce ciliary dysfunctions. TOCs were visually divided into ten equal sections, equivalent to 10% of ciliary activity per section. The average ciliary activity of ten tracheal rings / time point / group was calculated, as described in our previous study [30].

### Evaluation of lesion development via histopathological examination

TOCs were examined microscopically for histopathological changes to evaluate the lesion development after infection with the *M. gallisepticum* R variants. After fixation in 4% buffered formalin and embedding in paraffin wax, samples were cut into 2 µm thin sections. The sections were then transferred to microscope slides (corners cut, with a frosted edge, Carl Roth^®^ GmbH + Co. KG), air-dried for 24 h and subsequently stained with hematoxylin and eosin (H&E) following standard methods. The TOCs were evaluated for changes of the ciliated border, the epithelial cell morphology and in the submucosa, including ciliary destruction, loss of cellular integrity and oedematous changes.

### Examination of mucus production via Alcian blue staining

To examine if *M. gallisepticum* infection of TOC affects mucus production, visualization of acidic epithelial mucin via Alcian blue staining was performed. Samples were prepared as described above. The following procedure is based on the study from Crespo-Moral et al. [39] and was modified according to H&E staining procedures. Paraffin was removed, and samples were hydrated in xylene, descending alcohols, and distilled water. Afterwards, sections were stained with Alcian blue (Imperial Chemical Industries Ltd) for 20 min. After the last two washing steps, sections were dehydrated with an ascending alcohol series and mounted with Roti^®^Mount (Carl Roth GmbH + Co.KG). Goblet cells were counted for three microscopic fields (400-fold magnification), and the average number/ring was calculated. The group average was determined with five sections/group/time point.

### Statistical analysis

Data were tested for normal distribution with the Shapiro–Wilk Normality Test. Significant differences of normally distributed data between the groups at the same time points were evaluated with the Tukey HSD All-Pairwise comparison test (ANOVA, with α = 0.05) and verified by a two-group comparison with the two-sample t-test (*p* < 0.05). Significant differences of not normally distributed data between different groups at the same time points were evaluated by multiple comparisons with the Kruskal–Wallis Dunn all-pairwise comparison test (α = 0.05). Significances were verified by a two-group comparison with the Wilcoxon Rank Sum Test (*p* < 0.05) and adjusted with the Benjamini–Hochberg correction. All tests were conducted with Statistix, Version 10.0 (Analytical Software, Tallahassee, FL, USA).

### Experimental design

A total of six experiments was conducted to compare the infection pattern of *M. gallisepticum* encoding or not encoding for the proteins GapA and CrmA in TOC. In addition, the potential to induce histopathological lesions was determined for all *M. gallisepticum* variants. The following experiments in TOCs were addressed to the indicated objectives: 1^st^: examination of the bacterial adhesion-, gliding- and replication- pattern of the *M. gallisepticum* variants. 2^nd^: evaluation of lesion development after infection with the *M. gallisepticum* variants*.* 3rd: detection of alterations in the mucus production after *M. gallisepticum* infection. Unless otherwise stated, the following groups were included in all experiments: TOCs treated with pathogen-free organ culture medium and TOCs inoculated with either Rlow, Rhigh, RCL1 or RCL2. Each inoculum consisted of 1 × 10^3^ CFU/TOC in 100 µL 199 Hanks’ salts medium (*n* = 5 TOCs/treatment/group). TOCs were inoculated and incubated for 1 h at 37 °C. Afterwards, 900 µL pathogen-free organ culture medium, supplemented with 1% l-glutamine, 1% Penicillin G and 0.2% bovine serum albumin, was added. Medium was removed and replaced by fresh medium each 24 h. All experiments were conducted separately and repeated three times. The results represent summarised data from all repeated experiments.

## Results

A pre-experiment was conducted to determine the mycoplasma viability in an organ culture medium without TOCs. All four tested *M. gallisepticum* variants appeared viable in the medium supplemented with TOCs but died off without TOCs.

### The effect of gapA/crmA-modifications on the mycoplasma infection pattern

#### Examination of the bacterial cytadherence

**Figure 1:**
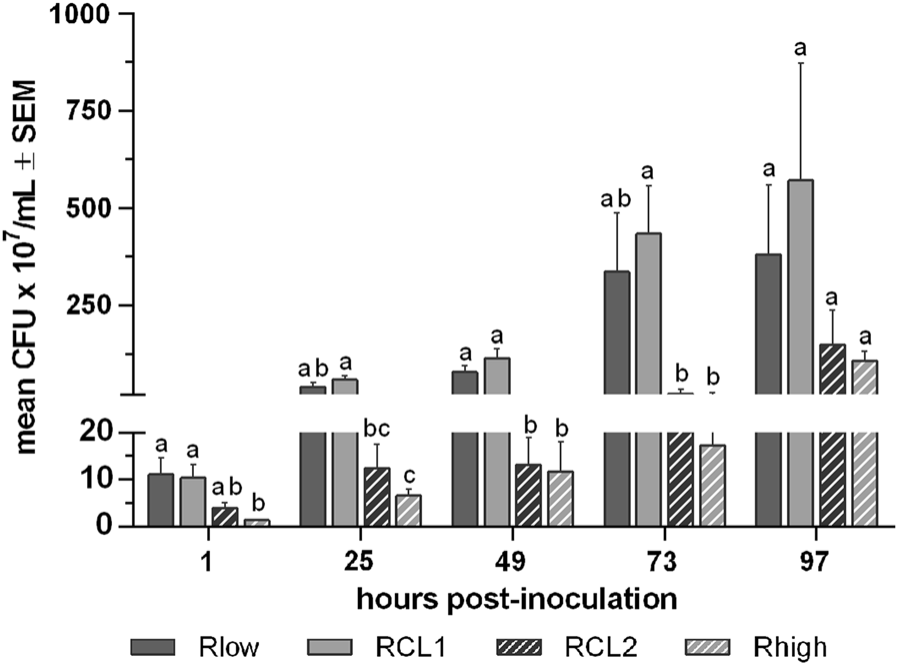
Effect of the *gapA*/*crmA* modifications on the cytadherence of *M. gallisepticum. M. gallisepticum* inoculated TOCs (*n* = 5/group/time) were transferred into HFLX medium at different time points after inoculation. TOC-adherent mycoplasma were incubated for 48 h in the medium. Medium was plated on agar plates and counted for colonies. Error bars indicate standard error of the mean (SEM). Different small letters indicate significant differences between *M. gallisepticum* variants at the same time point. One-way-AOV (ANOVA) Tukey HSD all-pairwise comparison test, with α = 0.05, verified with Two-sample t-test, *p* < 0.05. The control group remained *M. gallisepticum*-negative. Graph depicts data of one representative experiment.

To investigate the effect of *gapA*/*crmA* mutations on the adherence process, *M. gallisepticum* inoculated TOCs were transferred to HFLX medium after different time points. After 48 h of incubation, the medium was plated on Frey agar, CFUs were determined (Figure 1). The numbers of mycoplasma CFU/mL of medium containing bacteria dispersed from TOCs were comparable between the Rlow and RCL1 groups, as well as between the RCL2 and Rhigh groups. Significant differences in numbers of adherent mycoplasmas were detected from one to 73 hpi between Rlow/RCL1 and RCL2/Rhigh (*p* < 0.05).

### Detection of bacterial colonisation

**Figure 2:**
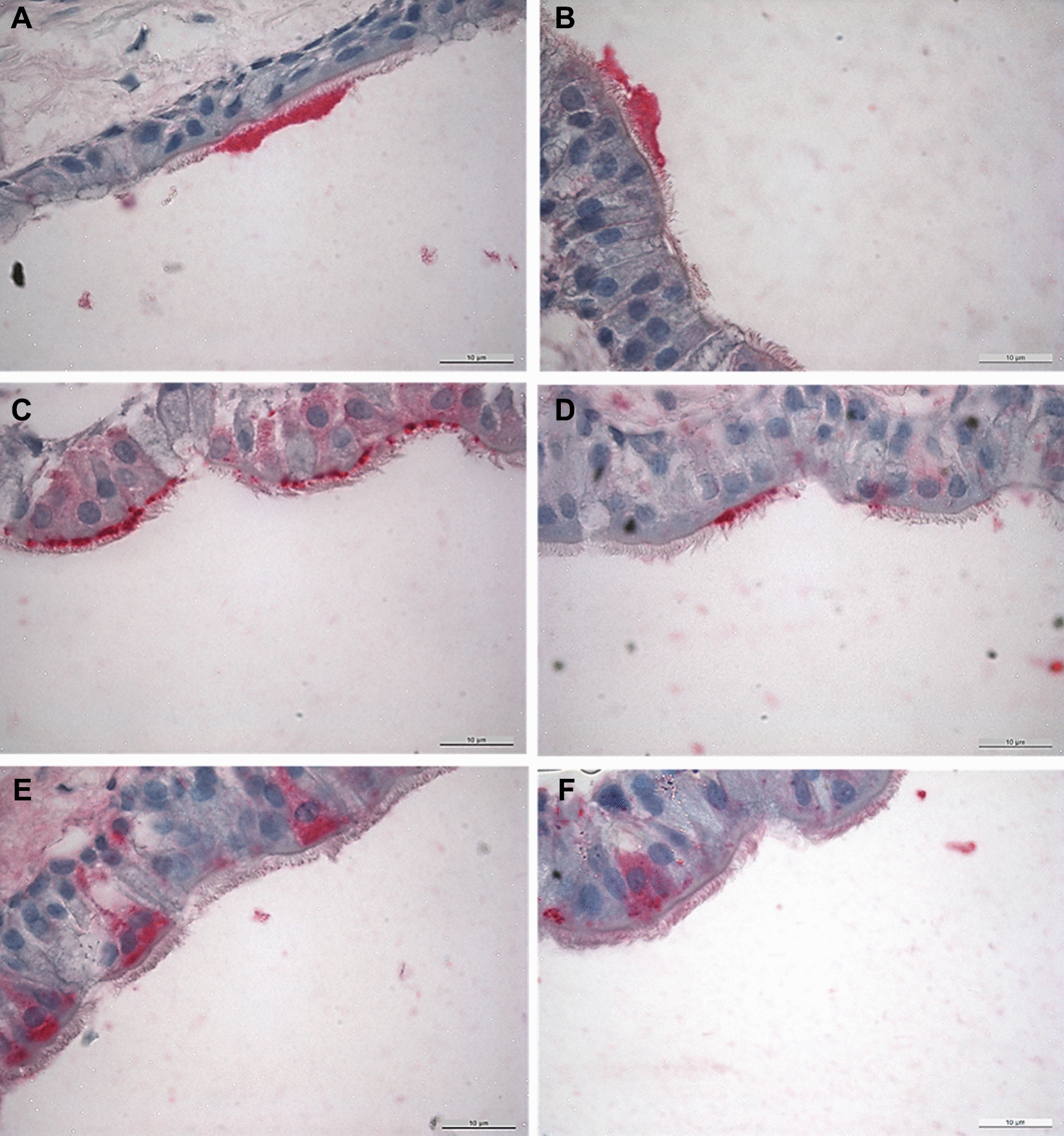
Effect of the *gapA*/*crmA* modifications on the distribution of *M. gallisepticum.* **A** RCL1 antigen attached to cilia tips, 1 hpi; **B** RCL2 antigen attached to cilia tips 25hpi; **C** RCL1 antigen attached to the apical cell membrane, 25 hpi; **D** RCL2 antigen attached to the apical cell membrane, 49 hpi; **E** Rlow antigen intracellularly located, 25 hpi; **F** Rhigh antigen intracellularly located, 73 hpi; all images at 1000 × magnification.

**Figure 3:**
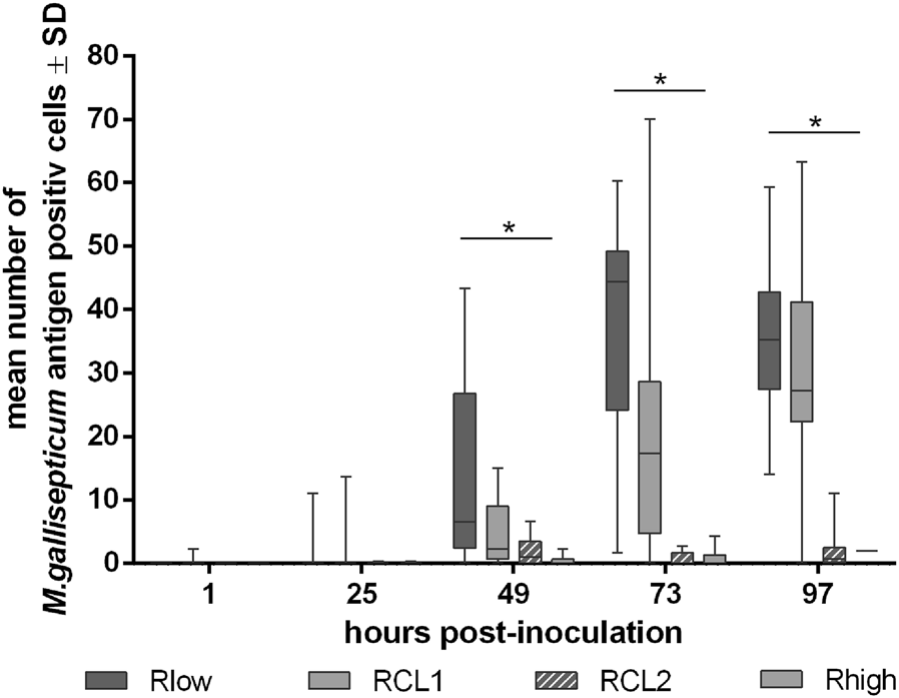
Effect of the *gapA*/*crmA* modifications on the colonisation of *M. gallisepticum.* TOCs (*n* = 5/group/time) were collected at different time points and processed for *M. gallisepticum* (*MG*)-antigen detection. Antigen-positive cells were counted in three microscopical fields per TOC. Asterisks indicate significant differences between Rlow/RCL1 and RCL2/Rhigh at the same time point. (**Kruskal–Wallis Dunn All-Pairwise Comparison Test, with α = 0.05, verified with Wilcoxon Rank Sum Test, *p* < 0.05. Graph represents summary of three independent experiments.

To show whether the *gapA*/*crmA* gene modifications affect the colonisation pattern of *M. gallisepticum*, TOCs were examined microscopically for antigen distribution (Figure 2) and further counted for *M. gallisepticum* antigen-positive cells (Figure 3). 1 h after inoculation, local accumulation of RCL1 antigen was detected at the cilia. Twenty-four hours later, the antigen of Rlow and RCL1 was observed over a large area on the apical cell membrane and intracellularly. In contrast, RCL2 and Rhigh antigen was found attached to the cilia tips at 24 hpi, and only sporadically on the apical border at 49 hpi. Intracellularly located antigen of RCL2 and Rhigh was detected at 73 hpi. Counting of antigen-positive cells revealed comparable increasing numbers of Rlow and RCL1 throughout the observation period (Figure 3). Meanwhile, the average of Rhigh and RCL2 antigen-positive cells remained significantly lower than in samples inoculated with Rlow and RCL1 (*p* < 0.05).

#### Quantification of bacterial replication pattern

**Figure 4:**
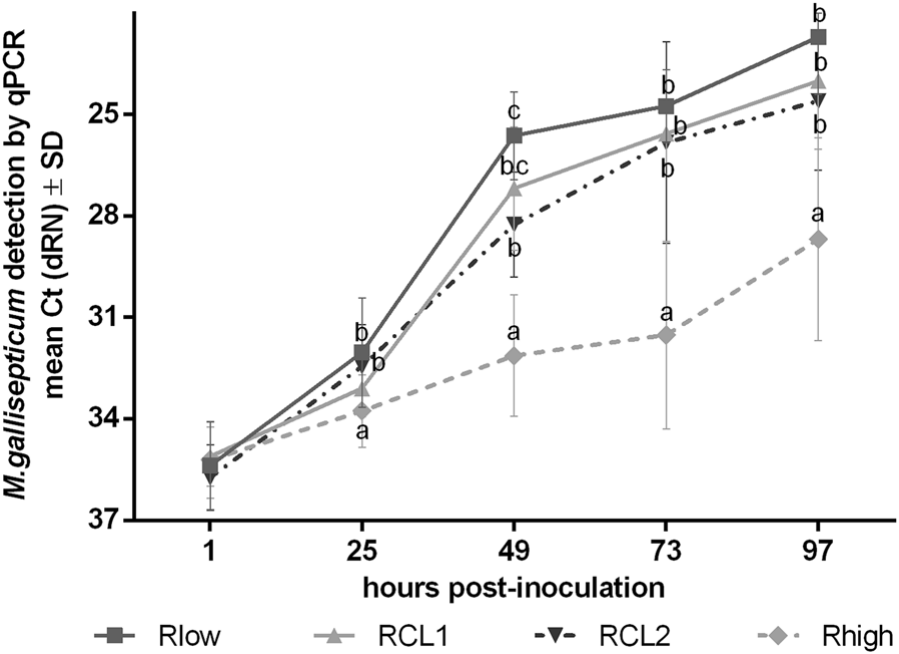
Effect of the *gapA*/*crmA* modifications on the replication of *M. gallisepticum.* TOCs (*n* = 5/group/time) were collected at consecutive time points and processed for *M. gallisepticum* (*MG*) genome quantification. Values were normalized against the RPL13 housekeeping gene. Normalized data are presented as mean Ct. Error bars indicated standard deviation (SD). Different small letters indicate significant differences between the four variants at the same time point. One-way-AOV (ANOVA) Tukey HSD all-pairwise comparison test, with α = 0.05, verified with Two-sample t-test, *p* < 0.05. Graph represents summary of three independent experiments.

We quantified the *M. gallisepticum* genome via qPCR to compare whether the *gapA*/*crmA* gene modifications affect the bacterial replication (Figure 4). The bacterial load of Rlow, RCL1 and RCL2 increased significantly from one to 49 hpi (*p* < 0.05), followed by a plateau-like phase from 49 to 97 hpi. At 49 hpi the RCL2 load was significantly lower, compared to Rlow (*p* < 0.05). The bacterial load of Rhigh showed a significantly lower increase than the other variants (*p* < 0.05).

### The effect of gapA/crmA-modifications on the ability of *M. gallisepticum* to alter the host mucociliary clearance

#### Observation for ciliary dysfunction

**Figure 5:**
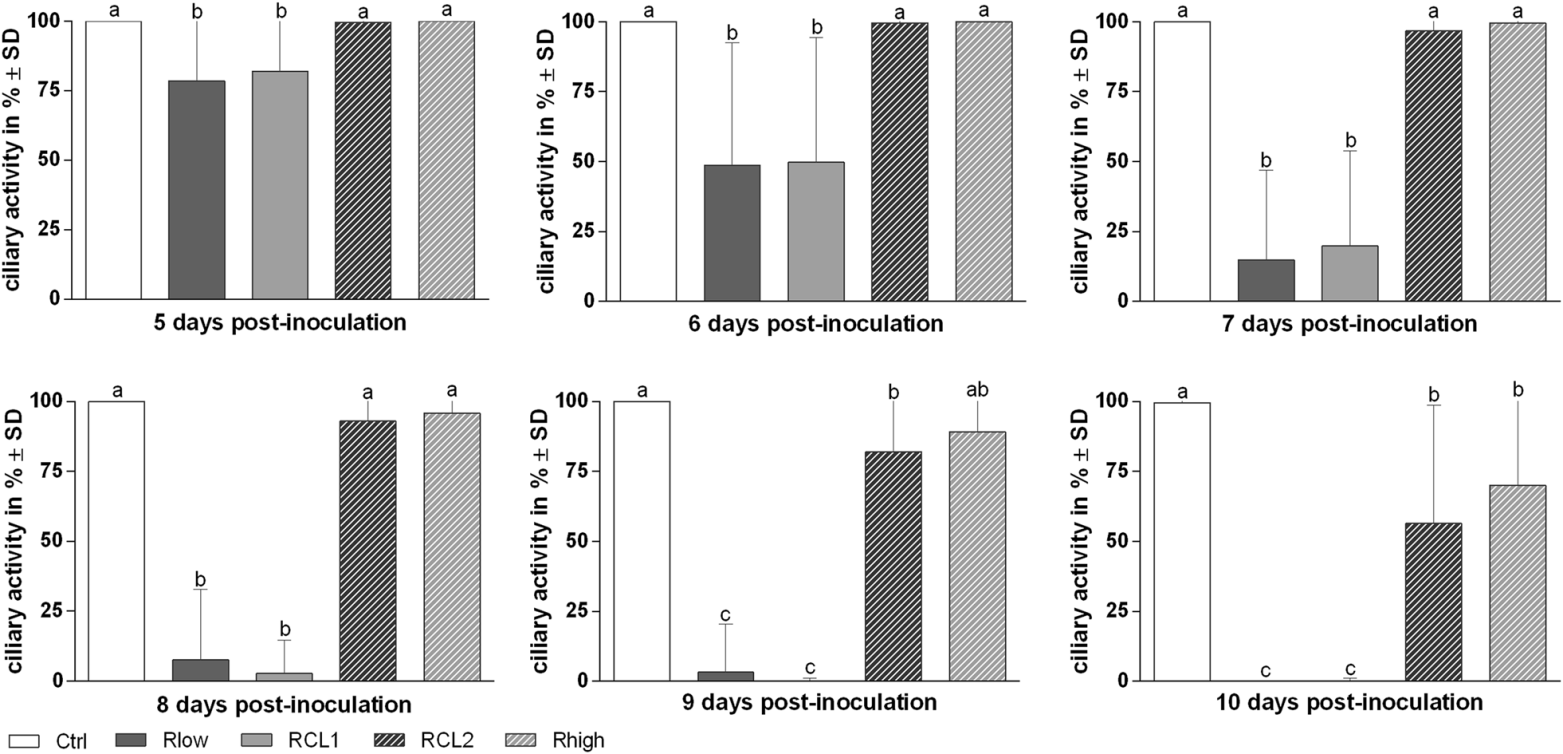
Ciliostasis-induction after inoculation with Rlow, RCL1, RCL2 and Rhigh. TOCs (*n* = 10/group) were microscopically evaluated for ciliary activity (%) over 10 days post-inoculation (dpi). Error bars indicate the standard deviation (SD). Different small letters indicate significant differences between different groups at the same time point. One-way-ANOVA Tukey HSD all-pairwise comparison test, with α = 0.05. No significant effect was detected from 1 to 4 dpi. Graphs represent summary of three independent experiments.

To investigate the impact of the four variants on the ciliary function, we microscopically observed the ciliary activity for 10 days (Figure 5). Rlow and RCL1 induced significant ciliostasis starting 5 dpi (*p* < 0.05). At 10 dpi, a total loss of ciliary activity for both variants was detected. Delayed by 4 days, RCL2 and Rhigh induced a significant loss of ciliary activity at nine and 10 dpi, respectively (*p* < 0.05).

#### Evaluation of the lesion development

**Figure 6:**
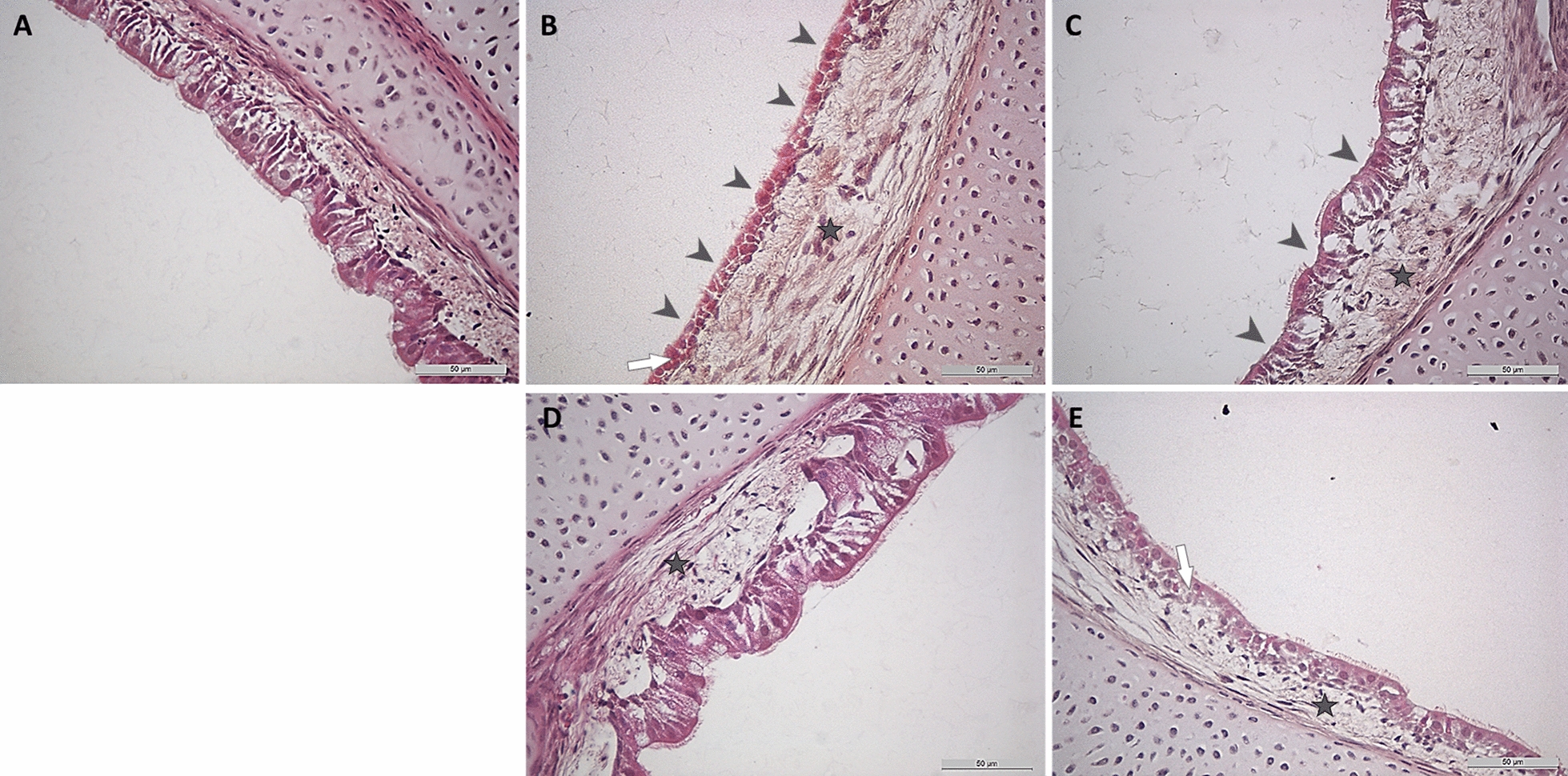
Lesion development in the tracheal epithelium after inoculation with Rlow, RCL1, RCL2 and Rhigh. Microscopic images of **A** control-, **B** Rlow-, **C** RCL1-, **D** RCL2- and **E** Rhigh-inoculated groups. All images represent the last time point of examination, 97 hpi 400-fold magnification. Grey arrowheads indicate ciliary loss, grey stars indicate submucosal edema, and white arrows indicate flattened, disintegrated epithelial cells.

After infection with the four *M. gallisepticum* variants, the ciliated border and epithelial compartments were microscopically evaluated to examine the histopathologic lesion development (Figure 6). The epithelial cells appeared flattened and disintegrated (Rlow and Rhigh groups), and the submucosa showed oedema at different degrees in all *M. gallisepticum* inoculated TOCs. Ciliary loss was detected in the Rlow group at 97 hpi and to a lesser extent in the RCL1 group. In contrast, no ciliary loss was detected in the RCL2-, and the Rhigh-inoculated groups throughout the observation period.

#### Detection and quantification of goblet cells

**Figure 7:**
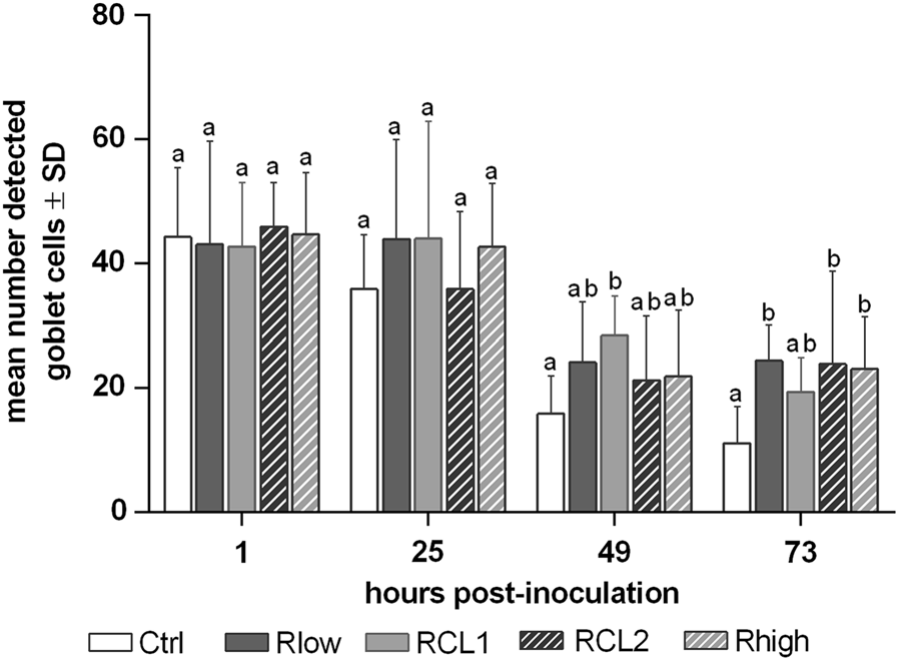
Detected goblet cells after inoculation with Rlow, RCL1, RCL2 and Rhigh. TOCs (*n* = 5/group/time) were collected at different time points and processed for goblet cell counting. Average of Alcian blue stained goblet cells of three microscopic fields was calculated. Error bars indicate standard deviation (SD). Small letters indicate significant differences between control- and *M. gallisepticum* variant groups at the same time point. One-way AOV (ANOVA) test, with α = 0.05, verified with two-sample t-test, with *p* < 0.05. Graph represents summary of three independent experiments.

**Figure 8:**
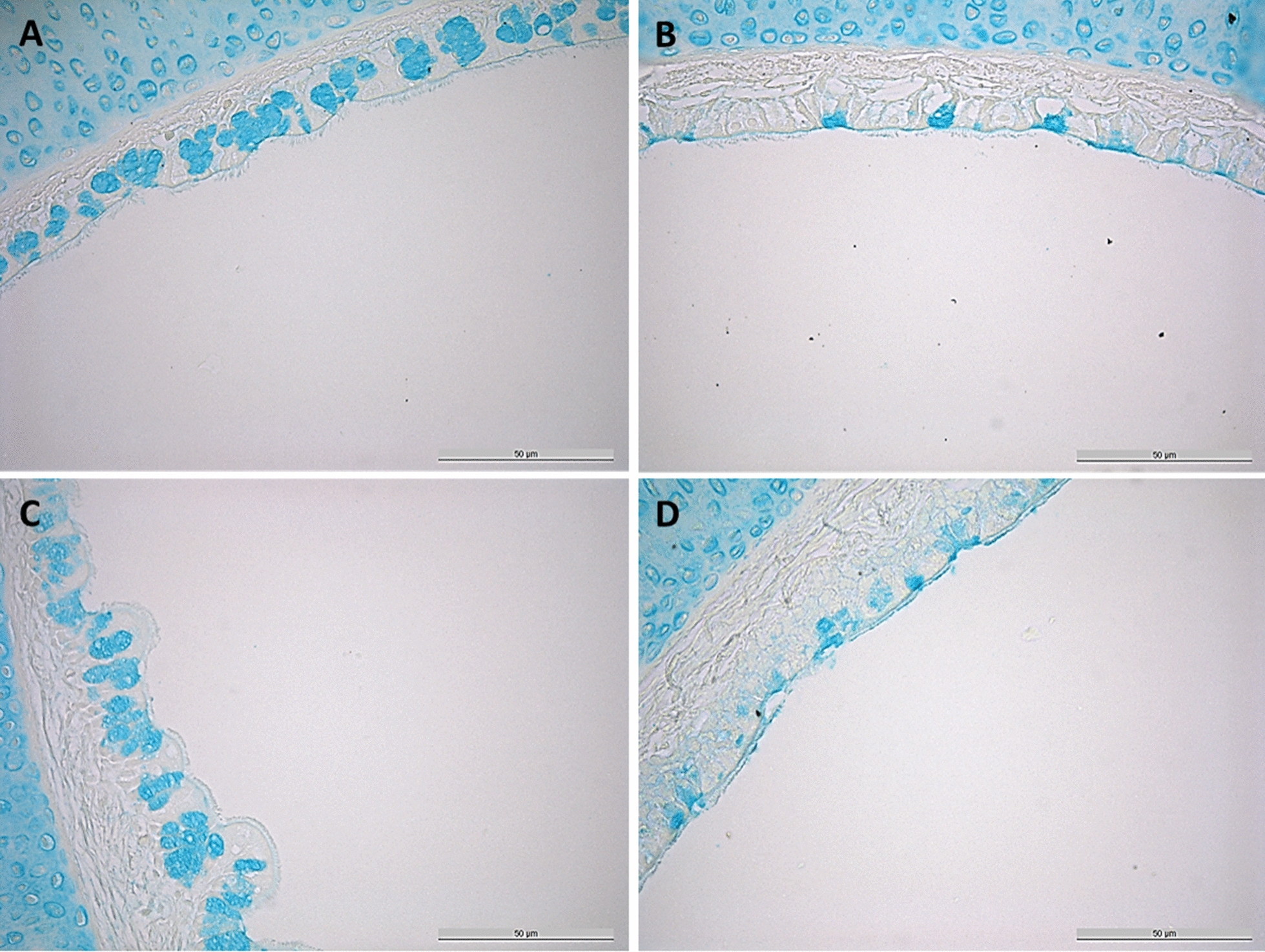
Goblet cell expression after inoculation with RCL1. Images represent samples stained with Alcian blue. Control **A** 1 hpi, **B** 73 hpi; RCL1 **C** 1 hpi, **D** 73 hpi; all images at 400-fold magnification.

To examine possible differences in mucus production between control- and inoculated TOCs, we evaluated the number of detectable goblet cells (Figures 7 and 8). The highest average number of visible goblet cells was detected at the first time point and decreased continuously from 1 to 73 hpi in the control- and the four *M. gallisepticum*-inoculated groups. At 49 hpi, the average number of detectable goblet cells in the control group tended to be lower than the *M. gallisepticum* groups, which was corroborated at 73 hpi (*p* < 0.05).

## Discussion

This study aimed to examine the *M. gallisepticum* strain R infection process at the mucosal surface of the trachea. It was hypothesised that the infection pattern is associated with vigorous bacterial adhesion and replication and subsequent lesion development, which correlates with the expression of the proteins GapA and CrmA. Four well-established *M*. *gallisepticum* strain R variants [12, 13, 17, 19, 24, 25, 27, 28] were used in this study to confirm this hypothesis. We inoculated TOCs with either low (Rlow) or high (Rhigh) passage variants, derived from the parental strain R, or the Rlow clonal variants RCL1 and RCL2, respectively. The GapA/CrmA-negative *M. gallisepticum* variants Rhigh and RCL2 lack the ability to form the terminal organelle (TO) [23], which consequently should lead to less efficient adherence to the TOCs. To evaluate the differences in adherence rates of *M. gallisepticum* variant*s* to the ciliated epithelial cells, we determined the number of TOC-adherent mycoplasmas. After *M. gallisepticum*-pre-inoculation of TOCs, they were transferred to the HFLX medium. The bacteria dispersed from the TOCs into the medium during 48 h of incubation. The medium was subsequently evaluated for CFU/mL. Significantly lower colony numbers of RCL2 and Rhigh were observed in the medium after detachment from the TOCs, compared to Rlow and RCL1 (*p* < 0.05). These findings may provide circumstantial evidence for more effective adherence to TOCs and colonisation of the epithelium by the GapA/CrmA-positive variants. However, different replication speeds of *M. gallisepticum* at transferred TOCs and the HFLX medium may also have contributed to the higher CFU of Rlow and Rhigh.

Whether there is a connection between adherence and gliding has been extensively analysed in previous studies with *M. pneumoniae* [14, 15, 20, 40, 41]. The P1 adhesion complex was demonstrated to be involved in both [22]. In this study, we determined the localisation and distribution of *M. gallisepticum* at the tracheal epithelium in 24 h intervals by histopathological examinations. Once attached to the cilia tips at 1 hpi, Rlow and RCL1 mycoplasmas moved to the apical cell border and, to some extent, invaded the cells within the next 24 h. This process was delayed by 24–48 h in the RCL2- and Rhigh inoculated TOCs. Interestingly, the GapA/CrmA-negative variants consistently accumulated only at few areas, in contrast to the diffusely distributed GapA/CrmA-positive variants. This resulted in a significantly higher number of antigen-positive cells of Rlow/RCL1 compared to RCL2/Rhigh throughout the observation period (*p* < 0.05). We clearly demonstrated that GapA and CrmA are necessary for efficient adherence and movement of *M. gallisepticum* from the cilia tips to the apical cell membrane, resulting in cell invasion. This supports the findings of Indikova et al. [13], who showed that GapA, CrmA and Mgc2 are involved in *M. gallisepticum’s* ability to glide*.* Colony satellite formation and high numbers of gliding tracks were observed for the motile variants Rlow and RCL1 using the qualitative microcinematography motility assay (MMA). In contrast, the “non-motile” variants formed satellite-less colonies and showed heavily reduced gliding paths.

Cytokinesis during the replication process is mediated by the initial TO moving away from subsequent TOs. Adhesion proteins are involved in both, movement, and formation of new TOs [14, 40, 42]. Therefore, it can be assumed that GapA and CrmA are involved in the replication process, as described for the *M. pneumoniae* P1 adhesion complex. Indeed, Rhigh shows a delayed and reduced dissemination in TOCs as confirmed by immunohistochemistry, as well as a significantly delayed and weakened increase of the bacterial load. That may indicate for less efficient replication than Rlow and RCL1. Furthermore, it was suggested that a single point mutation of *crmA* in RCL2, slightly different from wildtype (WT), may secure the survival compared to Rhigh, which has accumulated additional mutations affecting 29 ORFs, taking the intermediate position between Rlow and Rhigh [12, 17, 28]. In our TOC model, we confirmed the higher invasiveness of RCL2 compared to Rhigh by a significantly lower bacterial load of the latter. Whether the significant difference between RCL2 and Rhigh may additionally be caused by different reversion frequencies of the mutated genes [12] needs to be further elucidated.

Mycoplasmas are known to induce damage to the host cells, particularly ciliostasis, loss of cilia and alterations in the metabolic activities [10], allowing the continuation of the infection process. After treating hamster tracheal organ cultures with an *M. pneumoniae* protein extract, 70–100% loss of ciliary activity was observed, indicating the cytopathological effects of bacterial protein on host cells. Mycoplasma membrane-bound phospholipases may catalyse the hydrolysis of host cell phospholipids, which leads to adherence-induced oxidative damage of the cell [41]. Loss of tissue integrity and cell functions might also result from immune reactions to mycoplasma invasion, such as inflammatory responses or hydrogen peroxide production [36, 43, 44]. We found a faster progression of ciliary dysfunction in the TOCs infected with Rlow or RCL1 by 4 days, compared to a delayed effect induced by RCL2 or Rhigh. In addition, Rlow and RCL1 led to a diffuse or local loss of cilia, respectively, an oedematous submucosa and Rlow-caused flattened epithelial cells. No significant histopathological changes were detected in the RCL2 and Rhigh infected TOCs. Therefore, the initiation of cytopathological changes may be correlated with the expression of the GapA and CrmA proteins in *M. gallisepticum*. Our in vitro findings coincide with in vivo studies. The more virulent *M. gallisepticum* strain R induced massive destruction of tracheal cilia, in contrast to minor cilia lesions after inoculation with the low virulent *M. gallisepticum* strain F [45].

*M. pneumoniae* induces goblet cell hyperplasia and metaplasia (GCHM) and mucus hypersecretion in mouse lung tissue [9]. In human lung mucoepidermoid carcinoma (NCI-H292) cell lines and normal human bronchial epithelial (NHBE) cells, gene expression analysis revealed an *M. pneumoniae*-induced modulation of the STAT3-STAT6/EGFR-FOXA2 pathway. *M. pneumoniae* was suggested to suppress the expression of FOXA2, a repressor of mucin production, by activating the STAT6 and -3 pathways and EGFR signalling (inhibitors of FOXA5) and thereby inducing an increased mucus production [9]. In vivo co-infection studies with *M. gallisepticum* and subsequent *E.coli* revealed an activation of the IL-17 pathway and increased levels of inflammatory mediators, such as mucin-5 subtype AC (MUC5AC) [46]. In the TOC model, we observed a continuous decrease of detectable goblet cells in all *M. gallisepticum*-inoculated and control groups. The TOC-preparation procedures are known to cause the release of inflammation mediators, leading to an increase in the number of detectable goblet cells, which may still be detectable after 4 days of culture [46]. After that, they decreased again, but interestingly this process was delayed in *M. gallisepticum*-inoculated TOCs (*p* < 0.05), suggesting a modulatory effect of *M. gallisepticum* on goblet cell activity and mucus production, respectively, under in vitro conditions.

Overall, the tracheal organ culture model is an excellent tool to investigate *M. gallisepticum*-host interactions more closely. Comparing the four different *M. gallisepticum* variants, we demonstrated the important role of GapA and CrmA in the adhesion, gliding and replication process. Compared to low virulent RCL2 and Rhigh, the high virulent Rlow and RCL1 were more efficient in adhering to TOCs and epithelium colonisation, including faster movement from the cilia tips to the apical membrane and subsequent cell invasion. The less virulent variants RCL2 and Rhigh showed a more localised invasion pattern, which clearly is associated with fewer lesions, but interestingly not with a more differentiated mucus production than the more virulent Rlow and RCL1. Further studies are needed to elucidate the role of these important proteins individually and more closely, for example, by site-directed mutagenesis approaches.

## Data Availability

The datasets during and/or analysed during the current study available from the corresponding author on reasonable request.

## References

[1] Ferguson-Noel N, Armour NK, Noormohammadi AH, El-Gazzar M, Bradbury JM, Swayne DE, Bouliannea M, Logue C, McDougald LR, Naira V, Suarez DL (2020). Mycoplasmosis. Diseases of Poultry.

[2] Feberwee A, de Wit S, Dijkman R (2021). Clinical expression, epidemiology and monitoring of *Mycoplasma gallisepticum* and *Mycoplasma synoviae*: an update. Avian Pathol.

[3] El Gazzar M, Laibinis VA, Ferguson-Noel N (2011). Characterization of a ts-1-like *Mycoplasma gallisepticum* isolate from commercial broiler chickens. Avian Dis.

[4] Khalifa R, Eissa S, El-Hariri M, Refai M (2014). Sequencing analysis of *Mycoplasma gallisepticum* wild strains in vaccinated chicken breeder flocks. J Mol Microbiol Biotechnol.

[5] Rasoulinezhad S, Bozorgmehrifard MH, Hosseini H, Sheikhi N, Charkhkar S (2017). Molecular detection and phylogenetic analysis of *Mycoplasma gallisepticum* from backyard and commercial turkey flocks in Iran. Vet Res Forum.

[6] Razin S, Yogev D, Naot Y (1998). Molecular biology and pathogenicity of mycoplasmas. Microbiol Mol Biol Rev.

[7] Rosengarten R, Citti C, Glew M, Lischewski A, Droeße M, Much P, Winner F, Brank M, Spergser J (2000). Host-pathogen interactions in mycoplasma pathogenesis: virulence and survival strategies of minimalist prokaryotes. Int J Med Microbiol.

[8] Yiwen C, Yueyue W, Lianmei Q, Cuiming Z, Xiaoxing Y (2021). Infection strategies of mycoplasmas: unraveling the panoply of virulence factors. Virulence.

[9] Hao Y, Kuang Z, Jing J, Miao J, Mei LY, Lee RJ, Kim S, Choe S, Krause DC, Lau GW (2014). *Mycoplasma pneumoniae* modulates STAT3-STAT6/EGFR-FOXA2 signaling to induce overexpression of airway mucins. Infect Immun.

[10] Chandler DK, Barile MF (1980). Ciliostatic, hemagglutinating, and proteolytic activities in a cell extract of *Mycoplasma pneumoniae*. Infect Immun.

[11] DeBey MC, Ross RF (1994). Ciliostasis and loss of cilia induced by *Mycoplasma hyopneumoniae* in porcine tracheal organ cultures. Infect Immun.

[12] Indiková I, Much P, Stipkovits L, Siebert-Gulle K, Szostak MP, Rosengarten R, Citti C (2013). Role of the GapA and CrmA cytadhesins of *Mycoplasma gallisepticum* in promoting virulence and host colonization. Infect Immun.

[13] Indikova I, Vronka M, Szostak MP (2014). First identification of proteins involved in motility of *Mycoplasma gallisepticum*. Vet Res.

[14] Hatchel JM, Balish MF (2008). Attachment organelle ultrastructure correlates with phylogeny, not gliding motility properties in *Mycoplasma pneumoniae* relatives. Microbiology.

[15] Miyata M, Hamaguchi T (2016). Integrated information and prospects for gliding mechanism of the pathogenic bacterium *Mycoplasma pneumoniae*. Front Microbiol.

[16] May M, Papazisi L, Gorton TS, Geary SJ (2006). Identification of fibronectin-binding proteins in *Mycoplasma gallisepticum* strain R. Infect Immun.

[17] Papazisi L, Troy KE, Gorton TS, Liao X, Geary SJ (2000). Analysis of cytadherence-deficient, GapA-negative *Mycoplasma gallisepticum* strain R. Infect Immun.

[18] Mudahi-Orenstein S, Levisohn S, Geary SJ, Yogev D (2003). Cytadherence-deficient mutants of *Mycoplasma gallisepticum* generated by transposon mutagenesis. Infect Immun.

[19] Papazisi L, Frasca S, Gladd M, Liao X, Yogev D, Geary SJ (2002). GapA and CrmA coexpression is essential for *Mycoplasma gallisepticum* cytadherence and virulence. Infect Immun.

[20] Balish MF, Krause DC (2006). Mycoplasmas: a distinct cytoskeleton for wall-less bacteria. Microb Physiol.

[21] Henderson GP, Jensen GJ (2006). Three-dimensional structure of *Mycoplasma pneumoniae*’s attachment organelle and a model for its role in gliding motility. Mol Microbiol.

[22] Seto S, Kenri T, Tomiyama T, Miyata M (2005). Involvement of P1 adhesin in gliding motility of *Mycoplasma pneumoniae* as revealed by the inhibitory effects of antibody under optimized gliding conditions. J Bacteriol.

[23] Layh-Schmitt G, Harkenthal M (1999). The 40- and 90-kDa membrane proteins (ORF6 gene product) of *Mycoplasma pneumoniae* are responsible for the tip structure formation and P1 (adhesin) association with the Triton shell. FEMS Microbiol Lett.

[24] Papazisi L, Gorton TS, Kutish G, Markham PF, Browning GF, Nguyen DK, Swartzell S, Madan A, Mahairas G, Geary SJ (2003). The complete genome sequence of the avian pathogen *Mycoplasma gallisepticum* strain Rlow. Microbiology.

[25] Lin MY, Kleven SH (1984). Evaluation of attenuated strains of *Mycoplasma gallisepticum* as vaccines in young chickens. Avian Dis.

[26] Much P, Winner F, Stipkovits L, Rosengarten R, Citti C (2002). *Mycoplasma gallisepticum*: Influence of cell invasiveness on the outcome of experimental infection in chickens. FEMS Immunol Med Microbiol.

[27] Winner F, Rosengarten R, Citti C (2000). In vitro cell invasion of *Mycoplasma gallisepticum*. Infect Immun.

[28] Winner F, Markovà I, Much P, Lugmair A, Siebert-Gulle K, Vogl G, Rosengarten R, Citti C (2003). Phenotypic switching in *Mycoplasma gallisepticum* hemadsorption is governed by a high-frequency, reversible point mutation. Infect Immun.

[29] Arachchige SNK, Young ND, Shil PK, Legione AR, Condello AK, Browning GF, Wawegama NK, Palmer GH (2020). Differential response of the chicken trachea to chronic infection with virulent *Mycoplasma gallisepticum* strain Ap3AS and vaxsafe MG (Strain ts-304): a transcriptional profile. Infect Immun.

[30] Rüger N, Sid H, Meens J, Szostak MP, Baumgärtner W, Bexter F, Rautenschlein S (2021). New insights into the host-pathogen interaction of *Mycoplasma gallisepticum* and avian metapneumovirus in tracheal organ cultures of chicken. Microorganisms.

[31] Sid H, Hartmann S, Petersen H, Ryll M, Rautenschlein S (2016). *Mycoplasma gallisepticum* modifies the pathogenesis of influenza A virus in the avian tracheal epithelium. Int J Med Microbiol.

[32] Levisohn S, Dykstra MJ, Lin MY, Kleven SH (1986). Comparison of in vivo and in vitro methods for pathogenicity evaluation for *mycoplasma gallisepticum* in respiratory infection. Avian Pathol.

[33] Wise KS, Watson RK (1983). Mycoplasma hyorhinis GDL surface protein antigen p120 defined by monoclonal antibody. Infect Immun.

[34] Frey ML, Hanson RP, Andrson DP (1968). A medium for the isolation of avian mycoplasmas. Am J Vet Res.

[35] Albers AC, Fletcher RD (1982). Simple method for quantitation of viable mycoplasmas. Appl Environ Microbiol.

[36] Cherry J, Taylor-Robinson D (1970). Large-quantity production of chicken embryo tracheal organ cultures and use in virus and mycoplasma studies. Appl Microbiol.

[37] Sawada K, Agata K, Eguchi G (1996). Characterization of terminally differentiated cell state by categorizing cDNA clones derived from chicken lens fibers. Int J Dev Biol.

[38] Ferguson NM, Hepp D, Sun S, Ikuta N, Levisohn S, Kleven SH, Garcia M (2005). Use of molecular diversity of *Mycoplasma gallisepticum* by gene-targeted sequencing (GTS) and random amplified polymorphic DNA (RAPD) analysis for epidemiological studies. Microbiology.

[39] Crespo-Moral M, García-Posadas L, López-García A, Diebold Y (2020). Histological and immunohistochemical characterization of the porcine ocular surface. PLoS ONE.

[40] Krause DC, Balish MF (2001). Structure, function, and assembly of the terminal organelle of *Mycoplasma pneumoniae*. FEMS Microbiol Lett.

[41] Rottem S (2003). Interaction of mycoplasmas with host cells. Physiol Rev.

[42] Hasselbring BM, Jordan JL, Krause RW, Krause DC (2006). Terminal organelle development in the cell wall-less bacterium *Mycoplasma pneumoniae*. Proc Natl Acad Sci U S A.

[43] Rottem S, Naot Y (1998). Subversion and exploitation of host cells by mycoplasmas. Trends Microbiol.

[44] Takeuchi O, Kaufmann A, Grote K, Kawai T, Hoshino K, Morr M, Mühlradt PF, Akira S (2000). Cutting edge: preferentially the R-stereoisomer of the mycoplasmal lipopeptide macrophage-activating lipopeptide-2 activates immune cells through a toll-like receptor 2- and MyD88-dependent signaling pathway. J Immunol.

[45] Levisohn S, Yegana Y, Hod I, Herz A (1983). A correlative in vivo study of the surface morphology and colonisation of the chicken trachea infected by *Mycoplasma gallisepticum* strains R and F. Avian Pathol.

[46] Wu Z, Ding L, Bao J, Liu Y, Zhang Q, Wang J, Li R, Ishfaq M, Li J (2019). Co-infection of *Mycoplasma gallisepticum* and *Escherichia coli* triggers inflammatory injury involving the IL-17 signaling pathway. Front Microbiol.

